# The ‘Better Conversations with Primary Progressive Aphasia (BCPPA)’ program for people with PPA (Primary Progressive Aphasia): protocol for a randomised controlled pilot study

**DOI:** 10.1186/s40814-018-0349-6

**Published:** 2018-10-13

**Authors:** Anna Volkmer, Aimee Spector, Jason D Warren, Suzanne Beeke

**Affiliations:** 1 Division of Psychology and Language Sciences, Language and Cognition, University College London, Chandler House, 2 Wakefield Street, London, WC1N 1PF UK; 2 Division of Psychology and Language Sciences, Department of Clinical, Educational and Health Psychology, UCL, London, UK; 3 Department of Neurodegenerative Disease, Dementia Research Centre, UCL Institute of Neurology, London, UK

**Keywords:** Primary progressive aphasia, Conversation, Dementia, Speech and language therapy, Communication skills training

## Abstract

**Background:**

Primary progressive aphasia is a language-led dementia, often associated with frontotemporal dementia. It presents as insidious deterioration of language skills (e.g. naming objects and understanding complex sentences), with relative sparing of cognitive skills initially. There is little research examining the effectiveness of communication skills training for primary progressive aphasia, yet speech and language therapists (SLTs) report regularly using this in clinical practice. ‘Better Conversations with Primary Progressive Aphasia’ has potential to reduce barriers and increase facilitators to conversation and consequently improve confidence in communication and quality of life for people living with primary progressive aphasia and their conversation partners. The aim of this pilot study is to examine the feasibility of running a trial of the ‘Better Conversations with Primary Progressive Aphasia’ intervention.

**Methods:**

A single blind, randomised controlled pilot study will recruit 42 participants with primary progressive aphasia and their conversation partners across seven UK National Health Service Trusts. Participants will be randomised on a 1:1 basis, stratified by site, to receive either the ‘Better Conversations with Primary Progressive Aphasia’ intervention (21 couples) or no speech and language therapy treatment (21 couples). Participants are recruited by SLTs who will conduct pre-intervention assessment (week 1) and deliver the intervention (weeks 2 to 5). Junior researchers, who are blinded to allocation, will complete post-intervention measures (week 6). SLTs complete 9 h of training to prepare them to deliver the intervention. The primary objective of the study is to establish for a phase III effectiveness study whether the program can be delivered as intended in a UK National Health Service setting. Specifically, it will establish (1) the acceptability of randomisation, (2) an assessment of treatment fidelity to determine necessary levels of SLT training, (3) the most appropriate primary outcome measure, (4) sample size requirements, (5) predicted patient recruitment and retention rates and (6) refined inclusion criteria.

**Discussion:**

Insights from this study will be of relevance to guide development of future research and in particular, trials of therapeutic interventions in PPA, as well as for clinical care for this population.

**Trial registration:**

Retrospectively registered 28/02/2018 ISRCTN10148247

**Electronic supplementary material:**

The online version of this article (10.1186/s40814-018-0349-6) contains supplementary material, which is available to authorized users.

## Background

The prevalence of dementia is increasing, and it is anticipated that by the year 2050, there will be more than 131.5 million living with the diagnosis worldwide [1]. Primary progressive aphasia (PPA) describes a group of language-led dementias, often associated with frontotemporal dementia and considered the leading cause of dementia in people of working age [2, 3]. PPA initially presents solely as a language difficulty with impairments that superficially resemble classical stroke aphasia syndromes but worsening over time. There are three major PPA syndromes, namely semantic variant that affects the use and understanding of word meanings, logopenic variant that results in difficulties in word retrieval, and non-fluent agrammatic variant where individuals may present with either or both verbal dyspraxia and grammatical errors [3]. In many cases, other cognitive deficits (e.g. with memory) may not present for several years. As a result, people with PPA are often highly motivated to seek speech and language therapy. Perhaps, as a result of this retained awareness, people with PPA are prone to low self-esteem and poor confidence [4].

The research evidence for speech and language therapy interventions for PPA is sparse and is predominantly limited to naming therapies, word finding difficulty being a common impairment [5–11]. However, naming is often not the main limitation on communication function in PPA, and interventions are required to address real world communication function. This is particularly relevant given that prognosis in PPA is for loss of function, rather than improvement as in stroke aphasia, and there is a need to engage family/carers in speech and language therapy. Yet, there is a paucity of literature examining the impact that speech and language therapists (SLTs) can have in supporting a person with PPA and their family/carers with conversation [12, 13]. Nevertheless, SLTs in clinical practice report using communication training programs more often than naming therapies [14]. This is not surprising when reports suggest many patients disengage from naming therapies due to the frustration of practising individual words they will inevitably lose as the disease progresses [2].

SLTs have been reported to prioritise working on communication between people with PPA and their conversation partners (CPs), specifically targeting the use of meaningful strategies for both the person and their family members [15] using a variety of communication training approaches from the stroke and brain injury literature that have not been trialled for PPA [16]. Communication skills training has a growing evidence base in these fields [17, 18]. Programs have in common individualised feedback (often video-based) on facilitators (behaviours that help conversation such as using multi-modal communication—speaking, gesture, drawing and other communication aids) and barriers (behaviours that create problems in conversation such as the use of ‘test’ questions, asked by the CP despite already knowing the answer) to communication, followed by strategy training, with the aim of maximising the success of everyday conversations [19]. Whilst many such programs focus on training the CP only, Better Conversations with Aphasia (BCA) [20] aims to change the conversation skills of both a person with post-stroke aphasia and a CP [21–23]. BCA is a free online package consisting of a therapy manual and training materials for SLTs. Since its launch in 2013, it has attracted over 5000 users worldwide. BCA utilises video feedback to enable participants to reflect on communication facilitators and barriers, and the authors believe this to be a key mediator for improved conversation skill. In a UK-wide survey, a high proportion of SLTs report working with both the person with PPA and their CP and using BCA as a tool to support this therapy above other tools that only target the CP [16], thus motivating BCA as a target for adaptation to PPA over other possible alternative stroke aphasia programs.

In summary, the evidence base for communication training in PPA is limited, yet front-line SLTs favour this over naming programs with a stronger evidence base. This is because SLTs identify the need to support both a person with PPA and their CPs, who are equally distressed about conversation breakdown. We presently lack evidence to guide effective communication-based interventions in PPA. In response to this gap in the evidence base, the authors undertook a research study to develop and pilot a communication skills training program for people with PPA and their CPs. The BCA program for post-stroke aphasia was initially adapted to meet the needs of people with PPA using data collected from a UK-wide survey of speech and language therapy practice [16] and a systematic review of the research literature on functional communication focused interventions for people with PPA and their families [24]. It was further refined with SLTs who participated in a process using a nominal group consensus technique [25] to agree with the intervention objectives and with people with PPA and their families who took part in a series of focus groups. A BCPPA steering group (of people with PPA, their family members and expert professionals) was established at the start of the work to provide advice and feedback on all aspects of study management, including the co-production of materials for the program and support for future dissemination of results. In terms of the MRC guidance on development and evaluation of complex interventions [26], intervention refinement constituted phase I work to fully define BCPPA. This paper summarises the phase II randomised controlled pilot study protocol for the BCPPA program. Our UK-wide survey of SLTs [16] shows that there is no standard speech and language treatment for people with PPA, thus it is not possible to have a usual care group for the study. Instead, a no speech and language therapy treatment control group has been used. The protocol has followed the CONSORT (Consolidated Standards of Reporting Trials) guidelines and the SPIRIT (Standard Protocol Items: Recommendations for Interventional Trials) statement [27] as well as the TIDieR (Template for Intervention Description and Replication) checklist and guide [28].

### Aim

The primary aim of this study is to pilot the BCPPA program compared to a no speech and language therapy treatment control group over participating sites to establish for a main trial whether BCPPA can be delivered as intended in an NHS setting. Specifically, the aim of piloting the BCPPA program compared to a no speech and language therapy treatment control group is to establish:

- The acceptability of randomisation
- An assessment of BCPPA treatment fidelity to determine necessary levels of SLT training
- The most appropriate primary outcome measure
- Sample size calculation
- Predicted patient recruitment and retention rates
- Refined inclusion criteria

## Methods

### Design

**Fig. 1:**
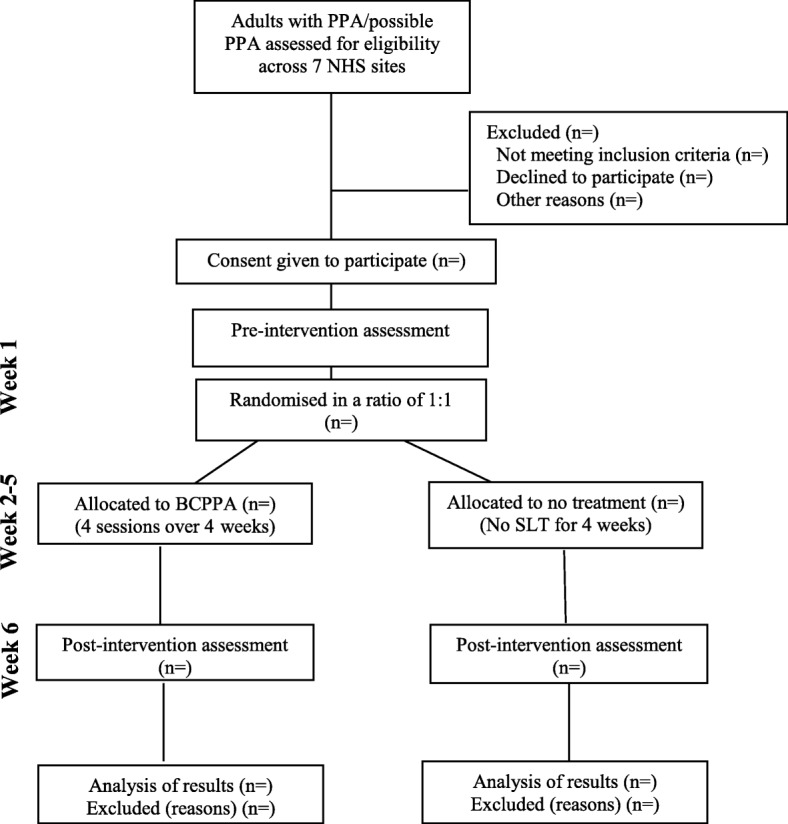
BCPPA participant flowchart through study

This is a single blind, randomised controlled pilot study with BCPPA treatment versus no speech and language therapy treatment. Participants will be involved for a total of 6 weeks: pre-intervention measures (week 1); intervention/control (weeks 2–5); and post-intervention measures (week 6). See Fig. 1 for participant flow chart through study.

### Setting

The seven participating NHS sites are located in England. Local collaborators (SLTs) at these sites will recruit participants, obtain consent, complete pre-intervention measures and deliver the BCPPA intervention across outpatient and community settings over a 20-month period.

### Population

The study includes adults (> 18 years) with a diagnosis or potential diagnosis of PPA (in view of the difficult and often protracted diagnostic process). See Table 1 for inclusion and exclusion criteria.

**Table 1:** Inclusion and exclusion criteria for potential participants

Inclusion criteria	Local collaborators at participating NHS sites will judge potential participants against the following criteria: (a) Have a diagnosis or possible diagnosis of PPA (b) Have some ability to communicate and understand communication in order to participate in the BCPPA program; (c) Are able to see and hear well enough to participate in the BCPPA program (d) Are functionally able to engage in the BCPPA program (i.e. able to maintain some concentration and remain in a 60–90 min session, minimal challenging behaviour that would be unlikely to cause disruption) (e) English as their language of daily use; (f) Have a conversation partner (CP) who is able to and consents to participating in the project.
Exclusion criteria	People will be excluded from participation in the pilot if they: (a) Have a history of brain lesions or major head trauma; (b) Have major physical illness or disability which could impact on participation; (c) Present with a major psychiatric diagnosis; (d) Present with prominent behavioural disturbance; (e) Present with prominent episodic memory, visual memory, or visuoperceptual impairments.

Note: The researchers acknowledge that the inclusion criteria may result in significant heterogeneity across participants—please refer to discussion of limitations

### Identification and recruitment of participants

**Fig. 2:**
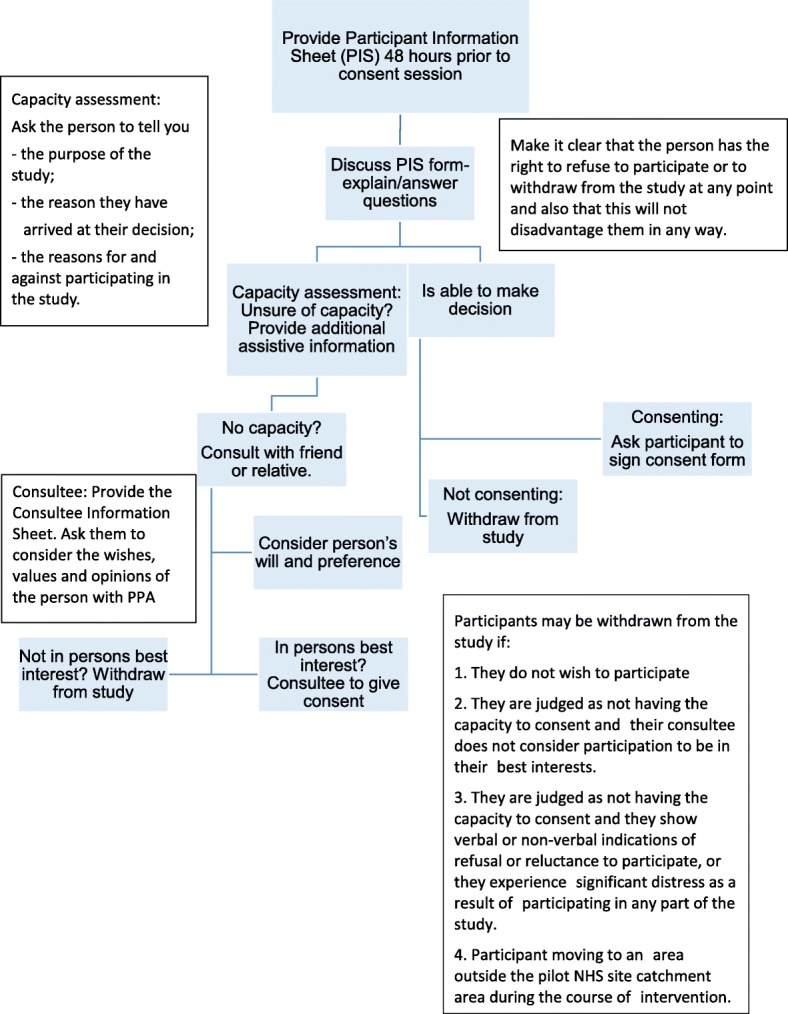
Consent flowchart

Local collaborators will be asked to identify people referred to their service who are suitable for the pilot using the inclusion and exclusion criteria (Table 1) and invite them to participate. Local collaborators will complete a log to record the number of people with a diagnosis of PPA who do not meet the inclusion criteria, they will also record the number of people who are eligible but who have declined to participate in the study and their reasons why if provided. People who meet the inclusion criteria will not be under any obligation to take part in this research and this will be made clear from the outset. Potential participants will be provided with a participant information sheet (see Additional file 1) before informed consent is obtained at least 48 h later (see Fig. 2 for consent flowchart).

All participants in the study will be in the mild to moderate stages of PPA and therefore would generally be expected to be competent to give informed consent to participate, provided that appropriate care is taken to explain the research and sufficient time is allowed for them to reach a decision. Due to the brief nature of the participants’ involvement in the study (5 weeks) it is not anticipated that decision-making capacity will change over this time; however, this will be monitored by the local collaborator who obtains consent following the Mental Capacity Act (MCA) [29], Royal College of Speech and Language Therapists (RCSLT) guidelines [30] and Good Clinical Practice Standards [31]. The local collaborators are specialist SLTs with considerable experience of supporting individuals with communication and cognitive impairment and who complete annual mandatory training on the MCA [29] and issues related to obtaining consent. If a local collaborator has any doubts regarding the capacity of a person with PPA to provide informed consent for this study, advice will be sought from the first author or an appropriate professional involved in their care, e.g. the GP, as is standard practice. Participant information sheets and consent forms (see the Additional files) have been designed to be dementia friendly [32] and modified with advice from the study’s steering group of people with PPA and their carers (Additional file 2).

### Randomisation

Randomisation will be conducted by the final author using a random number generator and stratified by site using blocks of four to balance across BCPPA treatment and no speech and language therapy treatment groups within each site. Block sizes will not be disclosed to local collaborators. Local collaborators will be informed of participant group allocation via email after pre-assessment has been completed.

### Blinding

Post-intervention measures will be administered by a pair of junior researchers (student SLTs at UCL) with skills in the assessment of people with PPA but crucially blinded to group allocation. Participants and family members will be asked not to reveal their allocation during the post-assessment session. They will be reminded of this prior to their appointment, by letter and verbally, at the start of the session. Should the students become unblinded during the reassessment process, this will be documented and the reasons recorded.

### Sample size justification

As there are no data available to estimate a sample size, the recruitment of participants has been dealt with pragmatically. Based on discussion with clinicians at the primary research site, it is estimated that it will be possible to recruit 42 participants over an 18-month period at seven sites. This pilot will provide information on recruitment and retention and facilitate a sample size calculation for a future full trial. Recruitment will be reviewed at 2-month intervals during the study, and the recruitment strategy will be amended as necessary to achieve the target number.

### Pre- and post-intervention measures

Participants will complete pre-intervention language, communication and quality of life measures (see Table 2) with the local collaborator in week 1 of the study. Completing assessment to gain an insight into areas of communication strength and difficulty prior to commencing intervention is routine procedure in speech and language therapy practice, and importantly, provides the opportunity for local collaborators to build rapport with participants before delivery of the intervention. All measures will be repeated after intervention at week 6 by a pair of junior researchers. In order to collect communication data, participants will be trained to independently use an iPad to video record conversation samples. A conversation topic list will be provided to support this process should they require it. Table 3 summarises the schedule of pre- and post-intervention measures.

**Table 2:** List of pre- and post-intervention measures

Language measures: 1. Comprehensive Aphasia Test [43] This language battery provides a profile of performance across all modalities of language production and comprehension.	
Communication measure: 1. Video recordings of conversation samples This informal measure adapted from the Aphasia Conversation Measure [21] identifies barriers and facilitators to conversation between the person with PPA and their CP and assesses changes in conversation after intervention	
Quality of life measures: 1. Dementia Quality of Life Measure [44] This questionnaire is designed to ask people with dementia to rate their quality of life across the three main domains of feelings, memory and everyday life. 2. The Aphasia Impact Questionnaire [45] A tool designed to measure the impact of living with aphasia across three domains of communication, participation and emotional well-being. 3. Communication Confidence Rating Scale for Aphasia [42] This is a questionnaire of communication confidence that uses a self-rating scale designed for people with aphasia. 4. Perceived Stress Scale [46] (completed by CP only) This is a self-report questionnaire for measuring the respondent’s perception of stress. 5. Zarit burden interview [47] (completed by CP only) This is a caregiver self-report questionnaire for measuring personal and care strain.

**Table 3:** Schedule of pre- and post-intervention measures

	Consent session			Preintervention assessment (1 week)	Treatment/control (4 weeks)				Postintervention assessment (1 week)				Final data collection
Week	0			1	2	3	4	5	6				7
Conversation	1a	2i	3i	4a					5a	6i	7i	8i	

Conversation video recording: a, assessor present but not in room; i, independent home recording

### Description of the intervention

#### BCPPA program

BCPPA provides a protocol for SLTs to deliver a four-session communication training program for people with PPA and their CPs. Participant's pre-intervention video-recorded conversation samples will be used to provide clips for video feedback during intervention sessions. The manual is made available to local collaborators online. The intervention is described in detail in Additional file 1, using the TIDieR checklist [28].

#### No speech and language therapy treatment

Those participants assigned to a no speech and language therapy treatment condition will receive usual healthcare provision (anticipated to include neurology, GP reviews, and allied health input such as physiotherapy). However, this will exclude speech and language therapy intervention for the duration of their participation in the study. The period of no speech and language therapy treatment will be 5 weeks – 4 weeks when the treatment group will receive BCPPA and 1 week when all participants complete post-intervention measures. As there is no critical period for treatment delivery for people with PPA, after this brief period, the participants allocated to the no speech and language therapy treatment group will resume all aspects of local speech and language therapy provision without further interruption.

### Training of local collaborators

Local collaborators will be trained to deliver the BCPPA program by the first author and will be provided with a training package including all required materials. Table 4 presents an overview of training content.

**Table 4:** Overview of training of local collaborators (SLTs)

Training goals For local collaborators to be able to: • Identify potential participants who meet the study inclusion criteria • Consent participants to the study • Deliver the BCPPA program • Complete pre-intervention measures • Complete fidelity measures • Access the support and advice of the researcher (first author) throughout the study	
Pre-training work • Pre-reading materials from the Study Training Pack to support participant identification, completion of outcome measures and delivery of intervention	
Day 1. (4.5 h) • Overview study procedures. • Discuss inclusion/exclusion criteria with vignettes to problem solve • Discuss consent procedures and flow chart with accompanying case study. • Discuss purpose and process of video recording conversation samples as outcome measure and to support therapy • Practice video recording for conversation samples. • Observe sample video recordings and identify barriers and facilitators to conversation with a view to planning therapy	
Day 2. (4.5 h) • Discuss and practice completing remaining outcome measures • Discuss therapy sessions with practical role play tasks, practice completing session plans, and observe sample video recordings demonstrating delivery of intervention, including goal setting and having emotional conversations. • Discuss fidelity measures (video recordings, local collaborator adherence questionnaire, and participant feedback questionnaires) • Provide contact information for ongoing support	

### Assessment of treatment fidelity

In order to ensure treatment fidelity, each local collaborator’s sessions with their first participant will be observed by the first author (via remote viewing of a video recording) and feedback will be provided over Skype or by telephone. After that, telephone and email support will occur as needed, to ensure knowledge and skills are maintained. In addition, to ensure adherence is maintained, local collaborators will be asked to audio record the intervention sessions with all participants. A random sample of 10% of recordings will be analysed using a treatment adherence checklist by two independent raters to permit an investigation of inter-rater reliability. A further 10% of this random sample will be analysed by a second rater, and any differences will be discussed and agreed. Local collaborators will also be asked to reflect on adherence by completing an adherence questionnaire and by documenting session length and tasks completed. These will be anonymously returned to the first author in pre-stamped addressed envelopes.

### Assessment of acceptability of the intervention

In order to ensure the treatment is acceptable to people with PPA, their CPs and local collaborators, each will be asked to provide feedback on the intervention. Accessible feedback forms will be given to participants with PPA and their CPs at every intervention session, to be completed and returned anonymously in pre-stamped addressed envelopes directly to the first author. Additionally, local collaborators will be asked to include feedback on acceptability as part of the adherence questionnaire, completed after every session.

### Data management

All personal information such as date of diagnosis, relevant medical and social history will remain confidential. Participants will be given a unique number which will be used on paperwork, assessment score sheets and in the names of all video files, and in all subsequent analysis documents and publications. Lists of participant names and their unique numbers (required to conduct the remote randomisation procedure) will be kept by a designated local collaborator in a locked cabinet at each NHS site. Each list will only contain the names of participants based at the relevant site.

Participants will consent to be video recorded in conversation for the purposes of outcome measurement and to provide clips for video feedback during intervention sessions. Participants’ faces will be fully visible in these video recordings as people’s expressions form a significant part of natural human communication, the focus of the intervention being piloted. Confidentiality can be guaranteed in the sharing of this footage at conferences and during teaching activities but not anonymity. Judicious selection of recordings will minimise this risk (e.g. footage where personal details are discussed will not be used, and names will be blanked out of the audio stream). Allied health professionals viewing this footage are bound by professional codes of ethics requiring them to maintain client confidentiality. Participants (and their CPs) will be asked whether they are willing to accept the possibility of being recognised, and can opt out of this use of their data whilst remaining part of the study. Only the research team members will have access to the entire video recorded data set. All transcripts of conversation data will be anonymised by the use of pseudonyms for all named people and places.

No data management committee will be established as it is felt that this short, small-scale pilot carries minimal risks. The study is compliant with General Data Protection Regulations. If information disclosed by any participant leads the first author to believe that a participant is at risk of harm or harming others, confidentiality will be broken to ensure safety.

### Data analysis

Analysis for this single blind, randomised controlled pilot study will involve both quantitative and qualitative methods. Language and quality of life assessment data will be entered into a database and analysed using the Statistical Package for the Social Sciences [33]. All data will be explored to test for assumptions of normalcy. If assumptions are not met, appropriate non-parametric tests will be used. Repeated measures ANOVA will be used in the analysis with group (no speech and language therapy treatment, BCPPA treatment) as a between subject factor and time (pre-therapy and post-therapy) as a within subject factor. This information will be used to determine a suitable sensitive outcome measure and to perform a power calculation to determine sample size for a fully powered trial. Conversation data will be analysed following a procedure developed for the BCA intervention [21] to identify change in the use of targeted strategies following intervention. This will involve counting barrier and facilitator behaviours in 5-min video samples selected from the pre- and post-intervention conversation samples. These counts will be analysed using Poisson Trend Test suitable for observations occurring in Poisson distribution. All pre-therapy conversations will be weighted the same as one another, as will all the post therapy conversations. Outcomes for different participants will be investigated using a Test for Homogeneity, where this is significant, and the effect for different dyads will be calculated using the Holm-Bonferroni procedure.

Descriptive statistics will be used to report recruitment, attendance, attrition, and reasons for dropout. A CONSORT (Consolidated Standards of Reporting Trials) and SPIRIT (Standard Protocol Items: Recommendations for Interventional Trials) [27] flow chart will be used to present overall recruitment to and progression through the study (Fig. 1). Recruitment and retention rates will be used to support sample size calculations to inform a future full trial and plan the required number of sites to meet this target. Adverse events will also be recorded, and participant feedback forms examined to inform future recruitment procedures. Stroke-related aphasia trials in the UK NHS have reported recruitment rates of between 38 and 44% from potential participants at the research sites [34–36].

Analysis of fidelity and adherence data will inform future training needs. Although fidelity data is sparse for speech and language intervention trials, processes such as those deployed in our study have been shown to achieve an average of 80–100% fidelity [37–39]. Thus, we have selected 80% as the minimum target for fidelity. Acceptability of the intervention will be considered, based on analysis of the participant feedback forms and adherence data to inform further refinements of the intervention prior to a future full trial. Analysis of participant feedback and adherence data will use descriptive statistics. For open-ended questions or ‘other’ response categories, qualitative analysis will be used, specifically identifying themes and sub-themes using thematic analysis [40].

### Criteria for success

This study will be considered appropriate to proceed to a full trial if:

- ▸Patients and local collaborators report generally positive views about acceptability of randomisation and of intervention as determined by evaluation of feedback forms;
- ▸A suitable sensitive outcome measure is determined and sample size estimated;
- ▸Local collaborator intervention-fidelity rate is at least 80%.

### Assessment and management of risk

This is a low-risk study. It is possible that participants will not experience improvements as a result of the intervention. However, there is evidence to suggest that such interventions are effective for improving communication and well-being in adults with non-progressive aphasia and their carers [14]. Importantly, there is no evidence to indicate that participants will experience any harmful effects. Most of the measures used in this study are frequently used in routine clinical practice with people with PPA. Additionally, the local collaborators are SLTs with skills to complete measures in a sensitive and supportive manner, minimising risk of distress. If participants do not feel emotionally or physically well enough to continue, then an assessment will be postponed. Video recording of conversations might also cause distress. To minimise the risk, we will ensure that as far as possible, the participants are familiarised with recording devices and understand why video recording is necessary. All procedures are set out within our participant information sheets (see Additional file 1) and consent forms, which were co-produced with the study steering group of people with PPA and their carers. Participants will be reminded that they can withdraw from the study at any time without giving a reason. Any adverse events will be recorded in the participant’s medical record, and the study sponsor informed.

## Discussion

To the best of our knowledge, this is the first randomised controlled UK pilot study of a conversation training intervention for people with PPA and their families. It will inform the feasibility of delivering a future full randomised controlled trial investigating the effectiveness of the BCPPA program in an NHS setting. There is little documented evidence of the impact of communication training for people with PPA and their CPs, yet there are reports of its widespread clinical use by SLTs across the UK [15, 16]. As in prior functional communication-focused PPA intervention studies [41], BCPPA might improve subjective confidence in communication (using the Communication Confidence Rating Scale in Aphasia [42] see Table 2 list of pre- and post- intervention measures) even if there is no clear impact on specific language measures, as well as potentially improving carer wellbeing. Thus, insights from this study will be of relevance to guide development of future research and in particular, trials of therapeutic interventions in PPA, as well as for clinical care for this population. The paucity of literature on functional communication-focused interventions for people with PPA makes the findings of interest to dementia researchers and SLTs, as well as people with PPA and their family members. The study will generate a unique dataset of language, conversation, and quality of life measures from people living with PPA and family members. It will lead to the identification of sensitive measures of the impact of functional communication-focused interventions for PPA. Additionally, the study will provide a rich source of information on acceptability of the BCPPA program to people with PPA and their CPs. Importantly, this study involves people with PPA and their families, and a group of expert SLTs, in the development of a manualised intervention program that meets their communication needs.

Study limitations include the inability to blind participants to group allocation, a common barrier in behavioural studies. Those allocated to the control group will not receive any intervention for 4 weeks as there is no comparable standard care intervention. Similarly, it is not possible to blind local collaborators delivering the intervention. For this reason, post-intervention assessment is not completed by the local collaborators but by pairs of junior researchers who will be blinded to group allocation. The inclusion criteria for this study may result in significant heterogeneity across participants in terms of language profile and communication difficulties, making it difficult to compare participants’ skills pre and post intervention. Thus, a range of outcome measures are being piloted across language, communication, and quality of life to identify the most sensitive across all participants. BCPPA is designed to be tailored to an individual’s needs, which may result in variation in the types of strategies identified and practiced. To counter this, the key components of BCPPA remain fixed, and measures of fidelity will demonstrate the consistency with which the intervention in delivered. A further potential limitation is ascertainment bias since access to speech and language therapy for PPA varies widely across the UK, and some of the likely barriers to access (socio-economic, cultural, linguistic, etc.) could also influence BCPPA outcomes. Despite these limitations, this study represents an important step toward a future full-scale RCT to determine the effectiveness of BCPPA for people with PPA and their family members.

The results of this study will be disseminated via presentations at national and international conferences and submitted for publication in peer reviewed scientific journals. In the medium term, the BCPPA program and training materials will be made available to SLTs via UCL’s public e-learning platform, alongside BCA [18]. With support from the study steering committee, results will be disseminated through professional and user group networks via publications and presentations, for example, at the PPA support group branch of the Rare Dementias Support Group based at UCL (http://www.raredementiasupport.org/).

### Trial status

Trial registration number ISRCTN10148247. Recruitment commenced in November 2017 and is due to end in March 2019.

## Additional files

Additional file 1: Description of BCPPA program (TIDieR). (DOCX 24 kb)

Additional file 2: Participant information sheet. (DOCX 2194 kb)

## Abbreviations

BCA: Better Conversations with Aphasia
BCPPA: Better Conversations with Primary Progressive Aphasia
CP: Conversation partner
ISRCTN: Numerical identification of randomised controlled trials
MRC: Medical Research Council
NHS: National Health Service
PPA: Primary progressive aphasia
RCSLT: Royal College of Speech and Language Therapists
SLT: Speech and language therapist
UCL: University College London

## References

[1] Prince M, Wimo A, Guerchet M, Ali GC, Wu YT, Prina M. World Alzheimer Report 2015 The Global Impact of Dementia. London; 2015. https://www.alz.co.uk/research/WorldAlzheimerReport2015.pdf. Accessed Apr 2018.

[2] Croot Karen, Nickels Lyndsey, Laurence Felicity, Manning Margaret (2009). Impairment‐ and activity/participation‐directed interventions in progressive language impairment: Clinical and theoretical issues. Aphasiology.

[3] Marshall Charles R., Hardy Chris J. D., Volkmer Anna, Russell Lucy L., Bond Rebecca L., Fletcher Phillip D., Clark Camilla N., Mummery Catherine J., Schott Jonathan M., Rossor Martin N., Fox Nick C., Crutch Sebastian J., Rohrer Jonathan D., Warren Jason D. (2018). Primary progressive aphasia: a clinical approach. Journal of Neurology.

[4] Volkmer A (2013). Assessment and therapy for language and cognitive communication difficulties in dementia and other progressive diseases.

[5] Graham KS, Pratt KH, Hodges JR (1998). A reverse temporal gradient for public events in a single case of semantic dementia. Neurocase.

[6] Jokel R, Rochon E, Leonard C (2006). Treating anomia in semantic dementia: improvement, maintenance, or both?. Neuropsychol Rehabil.

[7] Bier N, Macoir J, Gagnon L, Van der Linden M, Louveaux S, Desrosiers J (2009). Known, lost, and recovered: efficacy of formal-semantic therapy and spaced retrieval method in a case of semantic dementia. Aphasiology.

[8] Heredia CG, Sage K, Ralph MAL, Berthier M (2009). Relearning and retention of verbal labels in a case of semantic dementia. Aphasiology.

[9] Henry JD, Thompson C, Rendell PG, Phillips LH, Carbert J, Sachdev P (2012). Perception of biological motion and emotion in mild cognitive impairment and dementia. J Int Neuropsychol Soc.

[10] Jokel R, Graham NL, Rochon E, Leonard C (2014). Word retrieval therapies in primary progressive aphasia. Aphasiology.

[11] Rohrer JD, Knight WD, Warren JE, Fox NC, Rossor MN, Warren JD (2008). Word-finding difficulty: a clinical analysis of the progressive aphasias. Brain.

[12] Murray LL (1998). Longitudinal treatment of primary progressive aphasia: a case study. Aphasiology.

[13] Wong S, Anand R, Chapman S, Rackley A, Zientz J (2009). When nouns and verbs degrade: facilitating communication in semantic dementia. Aphasiology.

[14] Volkmer A, Beeke S (2015). How to help couples have better conversations. J Dement Care.

[15] Kindell J, Sage K, Cruice M (2015). Supporting communication in semantic dementia: clinical consensus from expert practitioners. Qual Ageing Older Adults.

[16] Volkmer A, Spector A, Warren JD, Beeke S. Speech and language therapy for primary progressive aphasia: referral patterns and barriers to service provision across the UK. Dementia. volume 0. pages 1–15. 10.1177/1471301218797240 PMC7309358 30180763

[17] Togher L, McDonald S, Tate R, Power E, Rietdijk R (2013). Training communication partners of people with severe traumatic brain injury improves everyday conversations: a multicenter single blind clinical trial. J Rehabil Med.

[18] Simmons-Mackie N, Raymer A, Cherney LR (2016). Communication partner training in aphasia: an updated systematic review. Arch Phys Med Rehabil.

[19] Simmons-mackie N, Savage MC, Worrall L. Conversation therapy for aphasia: a qualitative review of the literature. 2014;49:511–526. 10.1111/1460-6984.12097 24861277

[20] Beeke S, Sirman N, Beckley F, Maxim J, Edwards S, Swinburn K, Best W (2013). Better Conversations with Aphasia: an e-learning resource.

[21] Best W, Maxim J, Heilemann C, Beckley F, Johnson F, Edwards SI, et al. Conversation therapy with people with aphasia and conversation partners using video feedback: a group and case series investigation of changes in interaction. Front Hum Neurosci. 2016;10. 10.3389/fnhum.2016.00562. 10.3389/fnhum.2016.00562 PMC5097900 27872588

[22] Beeke S, Johnson F, Beckley F, Heilemann C, Edwards S, Maxim J (2014). Enabling better conversations between a man with aphasia and his conversation partner: incorporating writing into turn taking. Res Lang Soc Interact.

[23] Beeke S, Beckley F, Johnson F, Heilemann C, Edwards S, Maxim J (2015). Conversation focused aphasia therapy: investigating the adoption of strategies by people with agrammatism. Aphasiology.

[24] Volkmer A, Spector A, Beeke S. A systematic review of the research literature on functional communication focused interventions for people with PPA and their families. Int J Lang Commun Disord.

[25] Delbecq, A. L., Van de Ven, A. H., Gustafson DH. Group techniques for program planning : a guide to nominal group and Delphi processes. Glenview:Ilinois: Green Briar Press; 1975.

[26] Campbell M (2000). Framework for design and evaluation of complex interventions to improve health. BMJ.

[27] Chan A-W, Tetzlaff JM, Gotzsche PC, Altman DG, Mann H, Berlin JA (2013). SPIRIT 2013 explanation and elaboration: guidance for protocols of clinical trials. BMJ.

[28] Hoffmann TC, Glasziou PP, Boutron I, Milne R, Perera R, Moher D (2014). Better reporting of interventions: template for intervention description and replication (TIDieR) checklist and guide. BMJ.

[29] Health D of (2005). Mental Capacity Act.

[30] Royal College of Speech and Language Therapy. Information governance consent. https://www/rcslt.org.cq_live/resources_a_z/info_gov/info_gov_pdfs/igconsent. Accessed Feb 2017.

[31] Health Research Authority. Good Clinical Practice. https://www.hra.nhs.uk/planning-and-improving-research/policies-standards-legislation/good-clinical-practice/. Accessed Feb 2017.

[32] Pearl G. Engaging with people who have aphasia. A set of resources for stroke researchers. 2014. http://www.nihr.ac.uk/nihr-in-your-area/stroke/documents/Aphasia%20researcher%20resource.pdf. Accessed Feb 2017.

[33] IBM Corp. IBM SPSS Statistics for Windows. 2011.

[34] Bakheit AMO, Shaw S, Barrett L, Wood J, Carrington S, Griffiths S (2007). A prospective, randomized, parallel group, controlled study of the effect of intensity of speech and language therapy on early recovery from poststroke aphasia. Clin Rehabil.

[35] Palmer R, Enderby P, Cooper C, Latimer N, Julious S, Paterson G (2012). Computer therapy compared with usual care for people with long-standing aphasia poststroke: a pilot randomized controlled trial. Stroke.

[36] Bowen A, Hesketh A, Patchick E, Young A, Davies L, Vail A (2012). Effectiveness of enhanced communication therapy in the first four months after stroke for aphasia and dysarthria: a randomised controlled trial. BMJ.

[37] Heilemann C, Best W, Johnson F, Beckley F, Edwards S, Maxim J, Beeke S (2014). Investigating treatment fidelity in a conversation-based aphasia therapy. Aphasie und verwandte Gebiete 2.

[38] Whitworth A, Leitão S, Cartwright J, Webster J, Hankey GJ, Zach J (2015). NARNIA: a new twist to an old tale. A pilot RCT to evaluate a multilevel approach to improving discourse in aphasia. Aphasiology.

[39] Holland EJ, Watkins CL, Boaden E, Lightbody CE (2018). Fidelity to a motivational interviewing intervention for those with post-stroke aphasia: a small-scale feasibility study. Top Stroke Rehabil.

[40] Braun V, Clarke V (2006). Using thematic analysis in psychology Using thematic analysis in psychology. Qual Res Psychol.

[41] Rogalski Emily J., Saxon Marie, McKenna Hannah, Wieneke Christina, Rademaker Alfred, Corden Marya E., Borio Kathryn, Mesulam M.-Marsel, Khayum Becky (2016). Communication Bridge: A pilot feasibility study of Internet-based speech–language therapy for individuals with progressive aphasia. Alzheimer's & Dementia: Translational Research & Clinical Interventions.

[42] Babbitt EM, Heinemann AW, Semik P, Cherney LR (2011). Psychometric properties of the Communication Confidence Rating Scale for Aphasia (CCRSA): phase 2. Aphasiology.

[43] Swinburn KP, Gillian Howard D (2004). Comprehensive aphasia test.

[44] Mulhern B, Rowen D, Brazier J, Smith S, Romeo R, Tait R (2013). Development of DEMQOL-U and DEMQOL-PROXY-U: generation of preference-based indices from DEMQOL and DEMQOL-PROXY for use in economic evaluation. Health Technol Assess (Rockv).

[45] Swinburn K. Aphasia Impact Questionnaire-21. London: Connect.

[46] Cohen S, Kamarck T, Mermelstein R (1983). A global measure of perceived stress. J Health Soc Behav.

[47] Zarit SH, Orr NK, Zarit JM (1985). The hidden victims of Alzheimer’s disease: families under stress.

