# Supplementary material for: The ‘Better Conversations with Primary Progressive Aphasia (BCPPA)’ program for people with PPA (Primary Progressive Aphasia): protocol for a randomised controlled pilot study
Source: Pilot Feasibility Stud. 2018 Oct 13;4:158. doi: 10.1186/s40814-018-0349-6 (PMC6186039; doi:10.1186/s40814-018-0349-6)
Supplement: Supplementary file 1 — Description of BCPPA program (TIDieR). (DOCX 24 kb) [file 40814_2018_349_MOESM1_ESM.docx]

**TIDieR (Template for Intervention Description and Replication) Checklist for Better Conversations with Primary Progressive Aphasia (BCPPA)**

| Item Number | Item |
| --- | --- |
| 1. | **Brief name**  Better Conversations with Primary Progressive Aphasia (BCPPA) |
| 2. | **Why**  The goal of BCPPA is to improve communication between people with primary progressive aphasia (PPA) and their conversation partners (CPs), who are both involved in the training. This intervention is based on the techniques outlined in the Better Conversations with Aphasia program (BCA) and has been refined to meet the needs of people with PPA. The rationale is that by working with people with PPA and their partners, use of these jointly negotiated communication strategies will be maintained for longer as they target both the person with PPA and their partner who does not present with a progressive condition. Theory of behaviour change [1] has influenced the developed of BCPPA particularly; providing multiple opportunities for people to reflect on and practice strategies in therapy sessions and practice tasks, using video feedback to support participants to reflect on the impact of their communication on one another, focuses on elimination of barriers as well as implementation of facilitator strategies. BCPPA emphasises the joint setting of goals during therapy, facilitated by the speech and language therapist (SLT), thus implementing principles of self-management and self-efficacy [2] to set achievable and personally relevant goals. |
| 3. | **What**  Materials:  Each session is accompanied by a session plan, to guide the SLT, outlining specific goals and activities for the session. Participants pre-intervention video-recorded conversation samples will be used to provide clips for video feedback during intervention sessions. Handouts and homework sheets have been designed to accompany each session. The following provides a list of handouts and homework sheets used in the sessions:  Session plan 1: What is Conversation?   - Handout 1: How does conversation work? - Handout 2: What can go wrong in conversations? - Session 1 Homebased task 1: Your conversation troubles and repairs - Session 1 Homebased task 2: Strategies to help turntaking   Session plan 2: Goal Setting   - Handout 3: Goal setting - Handout 4: Strategies for the person with PPA - Session 2 Home based task 1: A chance to practice some strategies   Session plan 3: Practice   - Topic List Prompt Sheet - Session 3 Homebased task 1: A chance to practice some strategies - Session 3 Homebased task 2: Joining forces: Conversation strategy practice for both of you   Session plan 4: Problem solving and planning for the future   - Handout 5: Information for the future - Handout 6: Your strategies   Consider planning for future changes in communication  Training of intervention local SLT collaborators in the study employed the materials outlined above, alongside a series of video recordings of the first author delivering the intervention to a person with PPA and his conversation partner. These were used to support local collaborators in practice tasks using the session plans and choosing therapy materials for in the couple in the video examples. The video examples and session plans are available from the online BCPPA program which is hosted on the UCLeXtend website at <https://extend.ucl.ac.uk/> and is accessible to SLT collaborators involved in the pilot. In the medium term it will become freely available to all SLTs who register to use it. |
| 4. | **What**  Procedures: The person with PPA and their CP (a dyad) are provided with information and education on conversation (what it is and how it works). The dyad is then supported to reflect on a video sample of their own conversation to identify behaviours that facilitate or are a barrier to communication. Consequently participants are supported to set goals using the Goal Attainment Scales [3] to identify target communication strategies which they practise during activities, role play and homework tasks. Finally, the dyad is supported to plan for future communication changes. |
| 5. | **Who provided**  BCPPA is delivered by a qualified speech and language therapist (SLT), who has been trained using the online training modules accompanying the intervention program. |
| 6. | **How**  BCPPA is delivered face to face to a person with PPA and their chosen CP. |
| 7. | **Where**  Sessions are delivered at home or in an outpatient setting, depending on the location of speech and language therapy provision for PPA in a given NHS service. |
| 8. | **When and how much**  The BCPPA program consists of four 1.5 hour sessions delivered over 4 weeks. The following provides an overview of the aims of each intervention session:  Session 1: What is Conversation?   - Discuss aims of therapy - Discuss and explore what conversation is and how it can go wrong - Initial viewing of their own video   Session 2: Goal Setting   - Identify barriers and facilitators in their own conversation - Set goals for therapy based on this discussion   Session 3: Practice   - Practice conversation using the strategies identified during goal setting - Problem solve any issues that have arisen in using identified strategies in conversations outside of therapy sessions   Session 4: Problem solving and planning for the future   - Practice conversation using the strategies identified during goal setting - Consider planning for future changes in communication |
| 9. | **Tailoring**  BCPPA is tailored for an individual dyad (a person with PPA and their CP) by using video samples of their conversation recorded before intervention. These samples permit the SLT to assess the dyad’s communication difficulties and strengths before commencing the program. They are also used for video feedback to allow a dyad to set goals i.e. target communication strategies, which they practise during activities, role play and homework tasks. |
| 10. | **Modifications**  No modifications are anticipated. |
| 11. | **How well**  Planned: Adherence will be assessed via an anonymous questionnaire to local SLT collaborators following each session. Fidelity will be assessed by analysing a random sample of 10% of audio recordings of all intervention sessions for all SLT collaborators. |

1. Johnson FM, Best W, Beckley FC, Maxim J, Beeke S. Identifying mechanisms of change in a conversation therapy for aphasia using behaviour change theory and qualitative methods. Int J Lang Commun Disord. 2017;52:374–87. doi:10.1111/1460-6984.12279.

2. Yorkston K, Baylor C, Britton D. Incorporating the Principles of Self- Management into Treatment of Dysarthria Associated with Parkinson ’ s Disease. Semin Speech Lang. 2017;1:210–9.

3. Turner-Stokes L. Goal attainment scaling (GAS) in rehabilitation: a practical guide. Clin Rehabil. 2009;23:362–70. doi:10.1177/0269215508101742.
