# Supplementary material for: The ‘Better Conversations with Primary Progressive Aphasia (BCPPA)’ program for people with PPA (Primary Progressive Aphasia): protocol for a randomised controlled pilot study
Source: Pilot Feasibility Stud. 2018 Oct 13;4:158. doi: 10.1186/s40814-018-0349-6 (PMC6186039; doi:10.1186/s40814-018-0349-6)
Supplement: Supplementary file 2 — Participant information sheet. (DOCX 2194 kb) [file 40814_2018_349_MOESM2_ESM.docx]

**Participant Information Sheet**

Student Study

Better Conversations with Primary Progressive Aphasia (BCPPA)

**Pilot Study**

This project has been approved by the Health Research Authority. REC reference number: 17/LO/0357

**Before you accept it is important to understand:**

- **Who** is doing the research**?**
- **Why** is the research being done?
- **What** will it involve?
- **You can choose** if you do or do not want to do the research.
- You can **talk about this with family and friends**.
- You can **ask questions** at any time.

**Who is doing the research?**


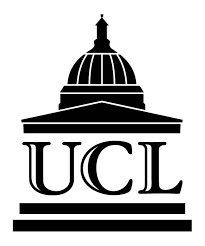


The research is being done from **University College London.**

The **main researcher on this project is**: Anna Volkmer

You can contact her on

**07879 655 426** or

[**a.volkmer.15@ucl.ac.uk**](mailto:a.volkmer.15@ucl.ac.uk)

The **National Institute for Health Research** is **paying** for this research


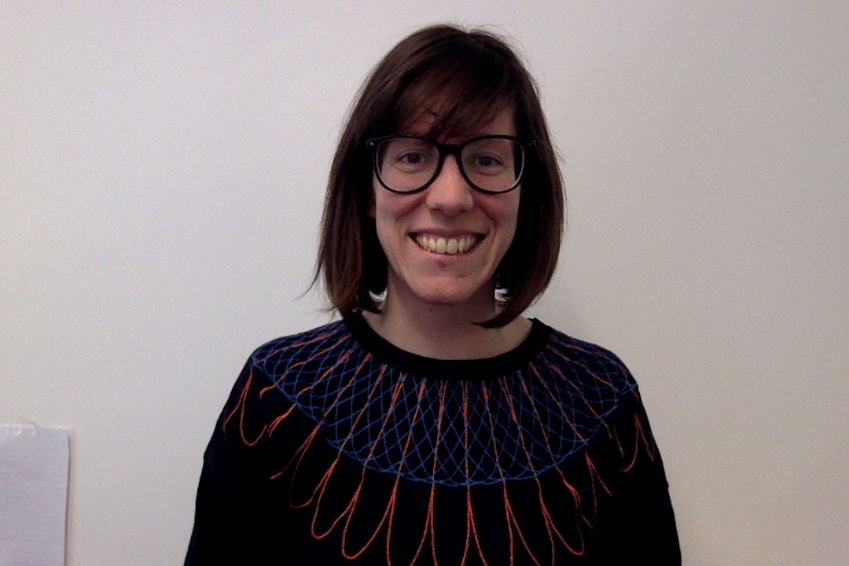

**Why is the research being done?**

Speech and language therapists provide **therapy** for people with **primary progressive aphasia**.

But we **don’t know** enough about it.

The research will help us **learn more**.

We are **developing a therapy treatment** called **Better Conversations with Primary Progressive Aphasia**.

The researcher will **look at the results**.

We want to know if it works.

We want to know if it works.

**What will it involve?**

We want **42 pairs** to take part.

We want to **compare** Better Conversations with Primary Progressive Aphasia with no treatment

**Half the people** will have the **Better Conversations with Primary Progressive Aphasia treatment** for **4 weeks.**

**Half the people** will have **no** **treatment** for **4 weeks.**

If you agree to take part **you will be randomly** assigned to either**:**

1. Better Conversations with Primary Progressive Aphasia

OR

1. No treatment.


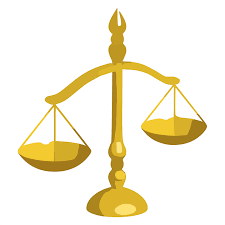


Better Conversations with PPA


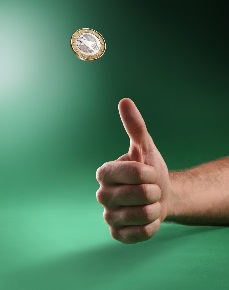


No treatment

**What will I have to do?**

**Week 6**

**Week 1**

**Weeks 2-5**

‘

The tests:

Your speech and language therapist will **test your talking, reading and writing.** She will ask you how you feel about talking and the impact that PPA has on your life.

You will be **video recorded 4 times** having a conversation with your family member. The speech and language therapist will make 2 videos, you will make 2 videos at home. **We will train you to use the video camera or iPad.**

GROUP A: Better Conversations with PPA therapy treatment:

You will **watch the videos** with the speech and language therapist.

The speech and language therapist will help you see the good strategies, and things that are not working well.

You and your family member will **practice** how to make **conversation easier**.

GROUP B: No treatment:

You will have **no speech and language therapy** for **4 weeks**. You will get all your other NHS care as normal.


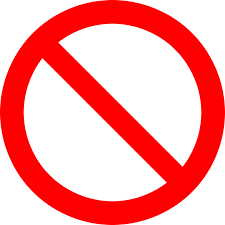


Repeating the tests:


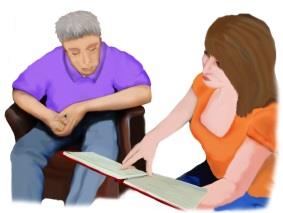


You will do **the tests of talking, reading and writing again.**

You will make **4 more videos** of you talking with your family member.

**Other questions?**

We will collect information about **you,** your **contact details** and when you were diagnosed with PPA. We will also ask you what other languages you speak, your previous occupation and other medical conditions you might have.

The information we collect will be stored in a locked filing cabinet at UCL for 10 years.

During the study the videos will be stored in a locked computer hard drive.

**After the 6 weeks of tests and treatment** is finished you can go back to your **normal speech and language therapy.**


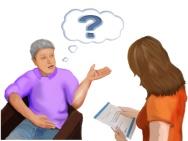

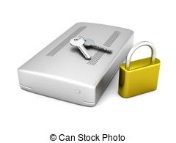

**Other questions?**

**You can choose** if you want to do the research or not.

You can **talk about this with family and friends**.

**You can stop** being in the research at any time

If you stop you do not have to give a reason…..and **you will still get your normal care**.

If you stop **your data will still be included** in the study.

**We will let your doctor know** that you are taking part (if you give permission).

If you tell us that you or someone else are **at risk of harm we will need to speak to other people** about this to keep everyone safe.

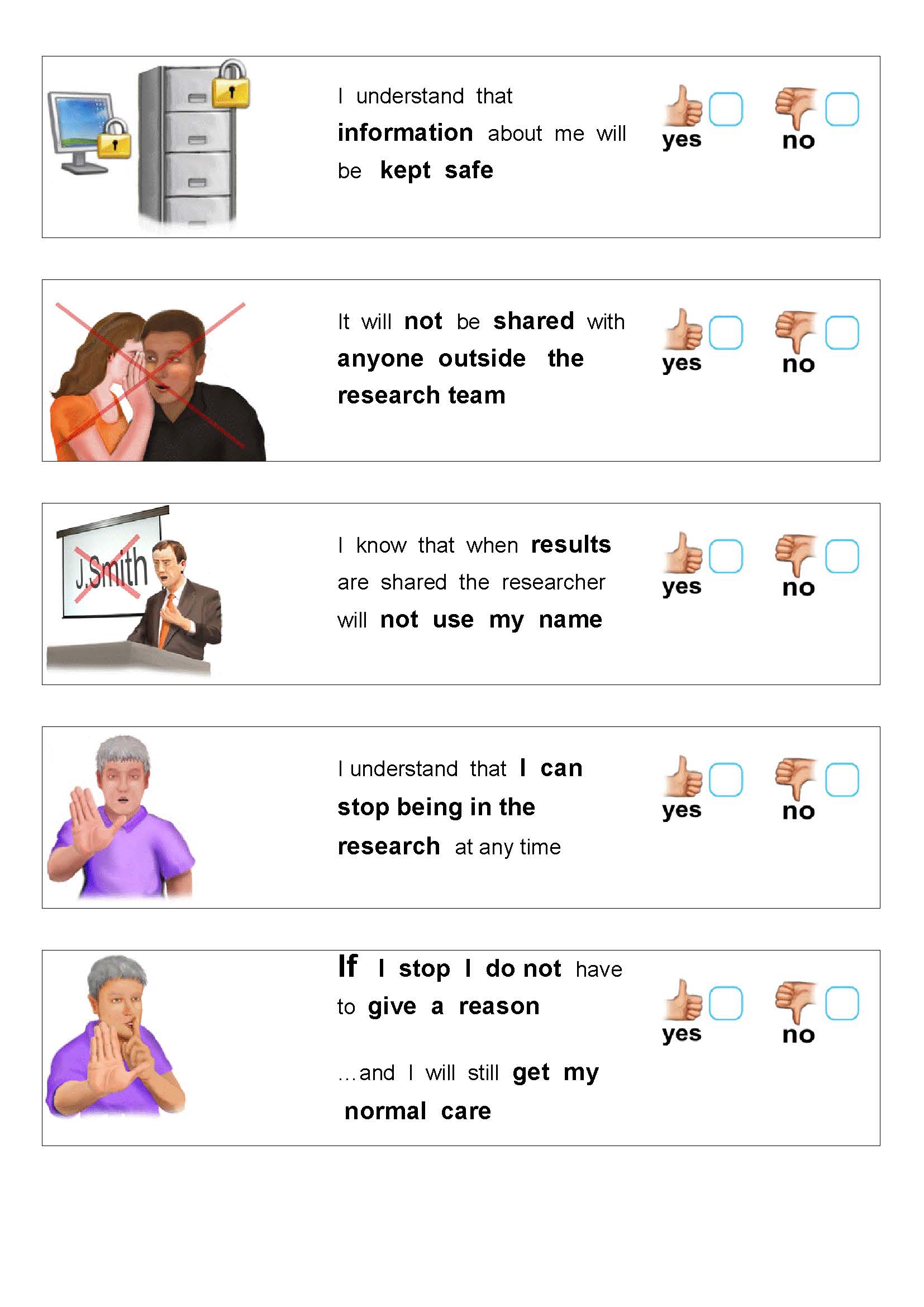


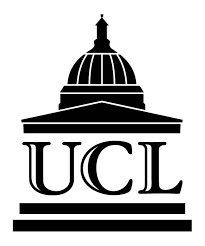


The videos will be stored in the Human Communication Audio Visual Archive (CAVA) at UCL Library for either

1. **As long as the Library exists**. If you agree your videos will be used **for future research**. Future researchers will sign a contract to respect your confidentiality, rights and dignity and use the videos responsibly.
2. **For one year after the study ends**, when they will be destroyed.

You can choose how long the videos are kept.

**Other researchers and students** might look at the videos to do more research.

They will be able to see your faces. They will keep the **videos** and the **information** about you **safe**.

**+++ Years**

**1 year**


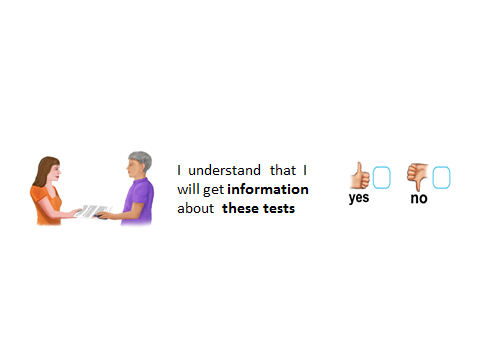


**What might be difficult about taking part?**

You may find **it won’t help you**.

You may find it **tiring**.

You may find it **upsetting**.

It will **take 6 weeks of your time**.

**6 weeks**

**What might be good about taking part?**

You may find it **helps** you.

Other people have found it:

- makes conversations easier
- improves things with their family members
- makes them feel more confident

You may **enjoy**  taking part.

You may find it **interesting**.

The results may **help people** in the future.

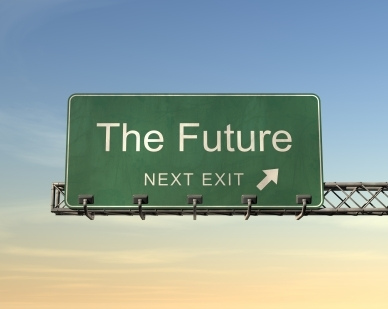


**
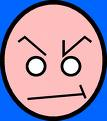
**

If you need to make a **complaint or you think you were harmed please contact**:

1. The main researcher,

Anna Volkmer,

Chandler House

2 Wakefield Street

London WC1N 1PF

**07879 655 426**

Email: [a.volkmer.15@ucl.ac.uk](mailto:a.volkmer.15@ucl.ac.uk)

Or

1. The project leader,

Suzanne Beeke

Chandler House

2 Wakefield Street

London WC1N 1PF

**020 7679 4215**

Email [s.beeke@ucl.ac.uk](mailto:s.beeke@ucl.ac.uk)

If you are **still** **unhappy**,

make a **formal complaint** by **writing** to the NHS Trust that is looking after you. Tell them the project number is …………………………….

You can also contact the **Patient Advice and Liaison Service (PALS)** in your local hospital trust. You can find your local PALS service by asking any local health provider or by looking on the NHS Choices website.

The University has **insurance.**
